# *De novo* transcriptome assembly and its annotation for the black ant *Formica fusca* at the larval stage

**DOI:** 10.1038/sdata.2018.282

**Published:** 2018-12-18

**Authors:** Claire Morandin, Unni Pulliainen, Nick Bos, Eva Schultner

**Affiliations:** 1 Organismal and Evolutionary Biology Research Programme, Faculty of Biological and Environmental Sciences, University of Helsinki, Helsinki, Finland; 2 Tvärminne Zoological Station, University of Helsinki, J.A. Palménin tie 260, FI-10900 Hanko, Finland; 3 Institut für Zoologie, Universität Regensburg, Regensburg, Germany

**Keywords:** Social evolution, Data acquisition, Transcriptomics, RNA sequencing, Entomology

## Abstract

Communication and nutrition are major drivers of fitness in ants. While communication is paramount to colony cohesion, nutrition is decisive in regulating reproductive division of labor among colony members. However, neither of these has been studied from a molecular perspective in developing individuals. Here, we report the availability of the first transcriptome resources for larvae of the ant *Formica fusca*, a species with excellent discrimination abilities and thus the potential to become a model system for studying molecular mechanisms of communication. We generated a comprehensive, high-coverage RNA-seq data set using Illumina RNA-seq technology by sequencing 24 individual 1^st^ - 2^nd^ instar larvae collected from four experimental groups (6 samples per treatment, 49 million mean reads per sample, coverage between 194–253×). A total of 24,765 unigenes were generated using a combination of genome-guided and *de novo* transcriptome assembly. A comprehensive assembly pipeline and annotation lists are provided. This dataset adds valuable transcriptomic resources for further study of developmental gene expression, transcriptional regulation and functional gene activity in ant larvae.

## Background & Summary

The evolution of insect societies represents a major evolutionary transition comparable to the evolution of multicellular organisms from free-living cells^1^. Just like multicellular organisms, insect societies consist of individual somatic units (sterile workers) and individual germ-line units (reproductive queens and males) that together form a highly integrated system. In contrast to multicellular organisms, insect societies can easily be broken down into their separate components (i.e. individuals), which can then be studied and manipulated in isolation. This makes insect societies excellent models for studying biological processes across all levels of biological complexity, from genomes to holobiomes and social groups^2^.

Among social insects, ants are especially numerous and diverse, with over 12,000 described species worldwide^3^. Studies of individual ants have typically focused on traits of adults, and in particular on the genetics, morphology, physiology and behaviour of reproductive queens and sterile workers^4^. Conceptual advances in biology, sparked by seminal work on the role of development in evolution^5^, are causing this focus to shift toward developing individuals. Ants are holometabolous insects and their development goes through four distinct stages: egg, larva, pupa and adult. While eggs and pupae do not participate directly in colony life, larvae are actively engaged in crucial colony-level processes (e.g. food processing) and represent the developmental stage during which growth and determination of reproductive caste, i.e. queen or worker caste, occurs. As a result, what happens during larval development invariably affects both individual traits such as caste, body size and fecundity, as well as key colony-level traits such as overall productivity and caste ratios^6^. To better understand the regulation of ant development as well as the complex behavioural and physiological interactions between larvae and adult colony members, high-quality genomic resources are necessary.

The focus of this study was on two features known to play a crucial role in insect sociality – communication and nutrition – both of which act as major forces driving fitness in ants. First, we were interested in how social interaction affects larval gene expression, in particular in the context of chemical communication. Communication between individuals is involved in many aspects of social life, including reproductive division of labor, brood care, foraging and colony defence, and is paramount to colony organization and cohesion^7,8^. Not surprisingly, chemical communication among adult ants has been extensively studied^8–11^. However, although larvae represents the life stage in which individuals are first exposed to complex social interactions^6^, and during which their discrimination abilities are most likely primed for adult life^12^, very little is known about how larvae participate in colony communication. Second, we were interested in the effect of nutrition on larval gene expression, as nutrition has been identified as one of the key factors involved in reproductive caste determination in wasps and bees^13–16^. While it seems clear that nutrition is also the most important factor regulating reproductive division of labor via its effects on queen-worker caste determination in ants^4,17^, the molecular basis of nutritional signalling has not been studied in individual larvae.

**Figure 1:**
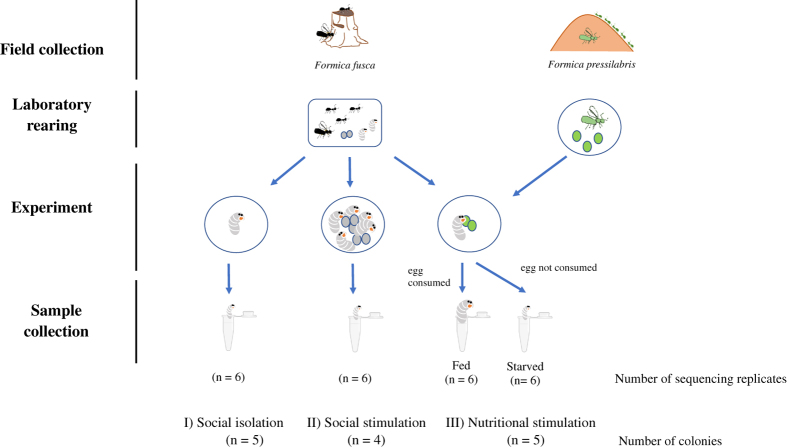
Flowchart of the experimental setup used to collect *F. fusca* larvae for sequencing showing treatment names and number of colonies.

**Figure 2:**
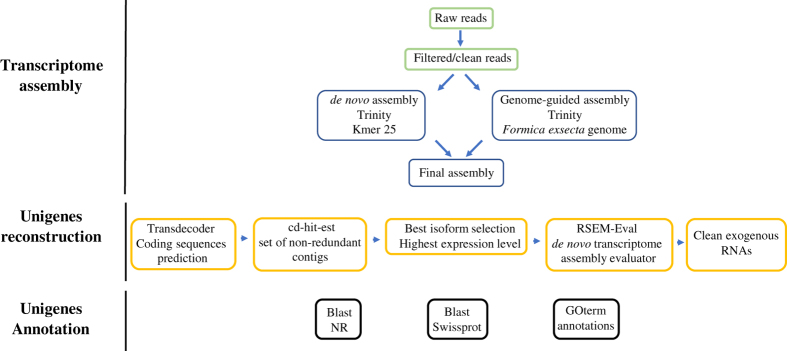
Flowchart of the RNA-sequencing setup and *de novo* transcriptome data analysis steps.

**Figure 3:**
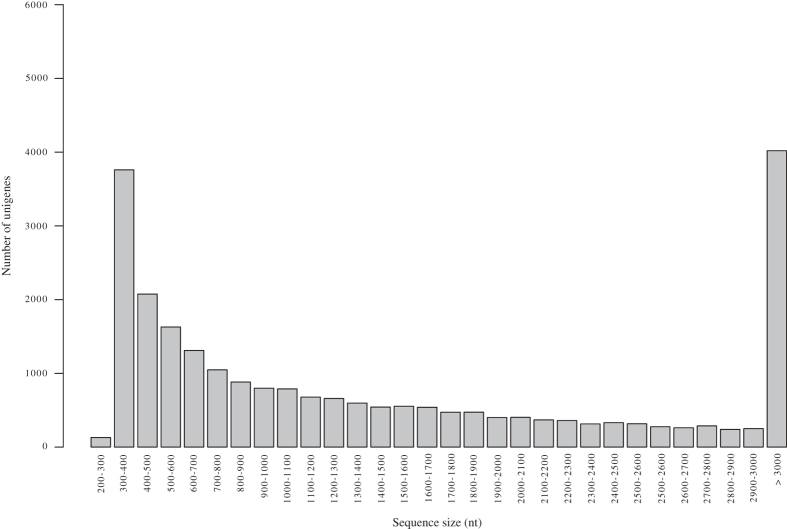
Length distribution of the final transcripts. The x-axis represents the length, the y-axis represents the number of transcripts.

This study reports the first transcriptomic sequences from whole larvae of the black ant *Formica fusca*, a common pioneer species in temperate climates^18^. As illustrated in Fig. 1, sequenced samples were subjected to four treatments: I) social isolation, II) social stimulation, IIIa) nutritional stimulation – “fed”, and IIIb) nutritional stimulation – “starved”. We provide a high-quality transcriptome assembly and annotated results, enabling comparisons with previously generated social insect larvae transcriptomes (e.g.^19^). The bioinformatics pipeline steps used in this study are shown in Fig. 2. We obtained a total of 24,765 assembled unigenes ranging in size from 19,880 bp to 297 bp, with a mean length of 1723,45 bp (Table 1, Fig. 3). Overall, this dataset adds valuable transcriptomic resources for further study of molecular correlates of development, transcriptional regulation and functional gene activity in ant larvae.

## Methods

### Experimental design

*Formica fusca* is a common pioneer species, which acts as a host to several temporary social parasite ant species^18^. Perhaps due to high parasite pressure, *F. fusca* workers and larvae exhibit precise discrimination abilities against con- and heterospecific individuals^20–25^, making it an ideal species to study the molecular correlates of communication. In addition, *F. fusca* larvae have been shown to consume both con- and heterospecific eggs^25,26^, showing that even larvae are able to discriminate friend from foe. This provides the unique opportunity to study how nutritional stimulation in the form of egg consumption affects developmental gene expression.

To address these questions, we collected whole colonies of *F. fusca* ants (n = 8) containing queens, workers and brood in the spring of 2016 from a known population on the Hanko peninsula in southwestern Finland in the vicinity of Tvärminne Zoological Station (59°54′46.3′N 23°15′55.9′E). After collection, colonies were transferred to Fluon^TM^ coated laboratory nest boxes, which were kept at room temperature. Colonies were provided with Bhatkar--Whitcomb diet^27^ and water daily. Shortly after collection, queens started laying eggs. Young larvae (1-3 days post hatching) were removed from colony fragments and visually size-matched according to head capsule width. Although the number of larval development stages in *F. fusca* is not known, related species exhibit three (*F. japonica*) or four (*F. polyctena*) larval instars^28^. Based on our visual inspection of larvae (Supplementary Figures S1, S2), only young, i.e. first or second instar larvae, were included in the experiments.

Each larva was then placed on a petri dish lined with humid sponge cloth, where it was subjected to one of three treatments for 24 h. In the first treatment, individual larvae were isolated without contact to other brood or adults (“social isolation). In the second treatment, larvae were kept with four other nestmate larvae and five nestmate eggs (“social stimulation”). By comparing gene expression of larvae from treatments one and two, we aim to elucidate how expression of candidate communication-related genes, in particular chemosensory and odorant-binding proteins^29,30^, is affected by the social environment larvae encounter. In the third treatment, individual larvae were kept with two *Formica pressilabris* eggs (“nutritional stimulation”). For this treatment, heterospecific eggs were chosen as a food source since larval egg consumption has been demonstrated previously in *Formica* ants^26^. Larvae that had consumed an egg after 24 h were designated as “fed larvae” while those that had refrained from egg consumption were designated as “hungry larvae”. By comparing gene expression of fed and hungry larvae, we aim to investigate how nutrition links to the expression of genes known to be implicated in nutrient-signalling (e.g. insulin signalling genes, TOR pathway genes) and identify genes potentially involved in nutrition-mediated caste determination processes^31^.

After 24 h, six larvae each from treatments one and two, and 12 larvae from treatment three (6 fed larvae and 6 hungry larvae) were collected in individual Eppendorf tubes containing 200 μL Trisure (Bioline) and stored at −80 °C until RNA extraction. For treatment two, only larvae that had not consumed any eggs or larvae were chosen for sequencing. For each treatment, larvae from four to five different colonies (out of 8) were chosen for sequencing to minimize effects caused by inter-colony variation. In addition, all treatments were set up in parallel over the course of 3 days to avoid any sampling effects.

### RNA extractions and library construction

Total RNA was extracted from the whole body of each individual larva using a standard Trizol protocol (TRIsure, Bioline). Subsequently, contaminating genomic DNA was removed by DNAse I digestion (Fermentas), and the RNA was purified using RNeasy MinElute kit (Qiagen), both following the protocol of the manufacturer. RNA was dissolved in 10 μL of milliQ water. The yield and quality of the RNA was verified by assessing the A_280_/A_260_ ratio (NanoDrop, Thermo Scientific) and inspected in a BioAnalyzer 2100 using RNA 6000 Nano kit (Agilent). Poly(A) RNA was isolated using the NEBNext Poly(A) mRNA Magnetic Isolation Module and libraries were prepared using the NEBNext Ultra Directional RNA Library Prep Kit for Illumina, both following the protocol of the manufacturer. The multiplexed samples were sequenced paired-end on five lanes of an Illumina HiSeq^TM^ 2500 2 × 100 bp (~400 M PE reads for each lane) at the FuGU lab in Helsinki, Finland.

### Transcriptome assembly

Approximately 2,640 million pairs of reads were generated using the Ilumina HiSeq^TM^ 2500 platform. The quality of raw reads was assessed with FastQC tools (http://www.bioinformatics.bbsrc.ac.uk/projects/fastqc) and MultiQC^32^. Raw reads were parsed through quality filtration (Trimmomatic^33^, options: LEADING:20 TRAILING:20 SLIDINGWINDOW:5:20 MINLEN:50). This program also searches for and removes any remaining TrueSeq Illumina adaptors in the reads. Unpaired reads were also discarded for the remainder of the assembly pipeline. After removal of low-quality reads, 2,383,894,158 clean reads (i.e. 90.3% of raw reads, Table 2) were retained and used in the transcriptome assembly pipeline described below. Genome-guided assembly usually provides high quality assembly of a reference transcriptome using the genome of a closely related species^34^. In order to recover a comprehensive set of transcripts, we used a combination of *de novo* assembly and genome-guided assembly known to give the best quality assembly^35,36^. We first performed a genome-guided assembly using Trinity software^37^ and the genome of the ant *Formica exsecta*, a closely related ant species (NCBI BioProject ID PRJNA393850 and BioSample: SAMN07344805). For this purpose, the trimmed reads were mapped to the *F. exsecta* genome using tophat software^38^. In parallel, we used the high-quality trimmed reads from all samples to perform a *de novo* assembly using Trinity software (trinityrnaseq-Trinity-v2.4.0) with 6 CPUS for the Inchworm and Butterfly steps, a maximum memory of 250 GB, a minimum t length of 200 bp, and the default K-mer of 25. The merged-assembly was built by combining the genome-guided and the *de novo* assembly. At this stage, a total of 412,776 transcripts were generated with a mean length of 1144.95 bp and an N50 of 2824 bp.

### Transcript reconstruction

We used TransDecoder *v*3.0.1 (TransDecoder. https://transdecoder.github.io/) to identify potential coding regions within the final assembly set of transcripts, following three steps. First, TransDecoder.LongOrfs was used to select the single best open reading frame (ORF) per transcript longer than 100 amino acids. In the second step, we identified ORFs with homology to known proteins using BLAST search (Uniprot database, BLAST version 2.2.26^+^, BLASTp, with an e-value cut-off ≤ 10^-5^) and searched for protein signature in the Pfam-A database. In the last step, the program TransDecoder. Predict uses this information to predict the coding sequences. A total of 189,123 coding sequences were kept for further analysis. To obtain a set of non-redundant transcripts, we then clustered highly similar coding sequences using cd-hit *v*4.6.5^39^ with an amino-acid sequence identity threshold of 0.99. A total of 61,036 coding sequences, belonging to 56,205 transcripts, were retained at this stage. Next, in order to obtain a set of unique, putative unigenes, transcripts containing these coding sequences were filtered to only retain the isoform with the highest expression, using the Trinity assembly information^34^. To do so, the cleaned reads were mapped to the cd-hit-filtered assembly using Bowtie2^40^ and the abundance of each transcript was estimated using RSEM^41^. A total of 28,808 unigenes were found. Thereafter, we used RSEM_EVAL package distributed with DENOTATE^42^ to assess the quality of the assembly. We filtered the assembly by applying RSEM-EVAL’s contig impact score, and 231 unigenes with impact scores less than or equal to zero were removed from the assembly, as recommended by Li *et al.*^42^. Finally, the unigene list was cleaned from probable exogenous RNAs known to be abundant in social insect *de novo* transcriptomes^43^. To this end, we used BLAST to compare the above list of unigenes against virus, fungal, protozoan and bacterial genome databases downloaded from NCBI (BLAST version 2.2.26^+^, BLASTn and BLASTp, with an e-value cut-off ≤ 10^−3^). A total of 3,812 unigenes that showed a minimum of 70% amino acid identity with at least one of the databases were removed from the assembly. The final assembly includes 24,765 unigenes.

### Transcriptome annotation and gene ontology

To provide comprehensive annotation of the final unigene set, we compared our final assembly to the NCBI non-redundant database (BLASTx with an e-value cut-off ≤ 10^3^). Unigenes were also searched against the Swissprot database (e-value cut-off ≤ 10^3^). A total of 19,688 unigenes returned a BLAST hit with the NR database (79.5%) and 13,129 (53%) with the Swissprot database. The annotations were submitted to the software Blast2GO (www.blast2go.com) to infer functional annotation and to obtain a list of gene ontology (GO) terms associated with the annotated genes. Of these unigenes, 11,898 contigs presented gene ontology (GO) annotation, with a mean GO level of 6.36 across biological process (BP), molecular function (MF) and cellular components (CC) categories (Supplementary Figures S3, S4). For biological process, 702 (5.9% of the number of sequences with GOterms) were in the oxidation-reduction process, 466 (3.7%) were in the proteolysis and 429 (3.6%) were in the regulation of transcription category. For cellular components, integral component of membrane represented the majority (3431, 28.6%), together with nucleus (755, 6.3%). For molecular function, 1104 (9.2%) ATP binding and 940 (7.84%) nucleic acid were highly represented (Supplementary Figures S5 (BP), S6 (MF), S7 (CC)). The number of unigenes annotated is shown in Table 1 and a complete list of BLAST and GOterm annotations are provided in Figshare (Annotation dataset, Data Citation 2).

## Data Records

The raw read files from this study were deposited to the DDBJ Sequence Read Archive (Data Citation 1). The database contains 24 records. For each treatment, six replicates were sequenced, using a single larva. Furthermore, the final assembly has been uploaded to figshare (Transcriptome assembly, Data Citation 2), and the final annotation dataset was also uploaded to figshare (Annotation dataset, Data Citation 2).

## Technical Validation

### Quality of the reads validation

**Figure 4:**
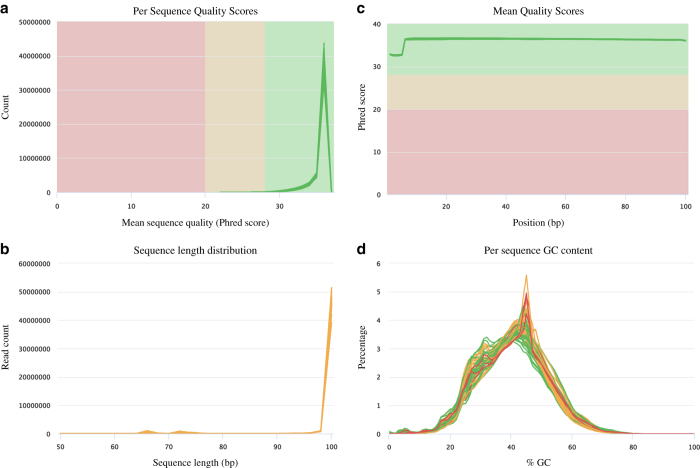
The cleaned reads from all 24 samples were assessed with FastQC and MultiQC. (**a**) Read count distribution for mean sequence quality. (**b**) Mean quality scores distribution. (**c**) Read length distribution. (**d**) Mean quality scores distribution.

To assess total data quality, we performed quality check using FastQC and MultiQC for all samples before and after adaptor/sequences trimming. The mean read counts per quality scores were higher than 35 (Fig. 4a). The mean quality scores in each base position were higher than 35 (Fig. 4b). The mean sequence lengths were 95-100 bp (Fig. 4c). The mean per sequence GC content was 40% (Fig. 4d).

### Transcriptome assembly validation

We ran the Trinity package utility script *TrinityStats.pl*, to obtain basic statistical information about the final assembly. The results of this evaluation are summarized in Table 1. Then, we quantified completeness of our final assembly by comparing the coding sequences of our final set of unigenes against a set of highly conserved hymenopteran single-copy orthologs using the BUSCO (Benchmarking Universal Single-Copy Orthologs) v2 pipeline^44^. The set of hymenopteran single-copy orthologs was downloaded from OrthoDB v9.1 database^45^. Following BUSCO recommendations, we calculated the number of complete/single-copy transcripts (2760, 62.5%), duplicated transcripts (1010, 22.9%), and fragmented transcripts (379, 8.6%). Only 266 (6%) single-copy orthologs were classified as missing from our final assembly. As one of the final steps, we ran RSEM Eval^42^ to evaluate our assembly and access how well the unigenes are supported by our RNA-Seq data. This step allowed us to filter unnecessary unigenes from our final dataset.

## Additional information

**How to cite this article**: Morandin, C. *et al*. *De novo* transcriptome assembly and its annotation for the black ant *Formica fusca* at the larval stage. *Sci. Data*. 5:180282 doi: 10.1038/sdata.2018.282 (2018).

**Publisher’s note**: Springer Nature remains neutral with regard to jurisdictional claims in published maps and institutional affiliations.

## Supplementary Material



Supplementary Figures

## Figures and Tables

**Table 1:** Properties and statistics of the final assembly of the *Formica fusca* larval transcriptome.

**Total unigenes**	**24765**
Percent GC	40.33
N50 (bp)	2868
Median contig length	1093
Average contig length	1723.45
Total assembled bases	42681228
N^o^ unigenes annotated in nr	19688
N^o^ unigenes annotated in Swissprot	13129
N^o^ unigene annotated in GO	11898

**Table 2:** Summary of the sequenced samples, number of reads and length, and sequencing depth.

Sample	Treatment	Combined number of reads (paired-end)	Combined length (bp)	Sequencing depth
UP1	Social isolation	88472546	8847254600	204
UP2	Nutritional stimulation – fed	96215472	9621547200	222
UP3	Social isolation	101706160	10170616000	235
UP4	Social isolation	101230178	10123017800	234
UP5	Nutritional stimulation – fed	102274664	10227466400	236
UP6	Nutritional stimulation – starved	98643690	9864369000	228
UP7	Social stimulation	101484884	10148488400	234
UP8	Nutritional stimulation – starved	105960956	10596095600	245
UP9	Nutritional stimulation – starved	99181338	9918133800	229
UP10	Nutritional stimulation – fed	100721650	10072165000	233
UP11	Social isolation	96545086	9654508600	223
UP12	Social stimulation	96197568	9619756800	222
UP13	Nutritional stimulation – starved	96320760	9632076000	222
UP14	Nutritional stimulation – fed	84069620	8406962000	194
UP15	Social isolation	99083672	9908367200	229
UP16	Social stimulation	93716720	9371672000	216
UP17	Social stimulation	102649030	10264903000	237
UP18	Nutritional stimulation – starved	96378826	9637882600	223
UP19	Nutritional stimulation – fed	100431728	10043172800	232
UP20	Social isolation	103424390	10342439000	239
UP21	Social stimulation	107013904	10701390400	247
UP22	Social stimulation	109412840	10941284000	253
UP23	Nutritional stimulation – fed	102714942	10271494200	237
UP24	Nutritional stimulation – starved	100043534	10004353400	231
