# Supplementary material for: De novo transcriptome assembly and its annotation for the black ant Formica fusca at the larval stage
Source: Sci Data. 2018 Dec 18;5:180282. doi: 10.1038/sdata.2018.282 (PMC6298252; doi:10.1038/sdata.2018.282)
Supplement: Supplementary Figures [file sdata2018282-s2.pdf]

Supplementary File 1

Supplementary Figure S1 .... Page 2

Supplementary Figure S2 .... Page 3

Supplementary Figure S3 .... Page 4

Supplementary Figure S4 .... Page 5

Supplementary Figure S5 .... Page 6

Supplementary Figure S6 .... Page 7

Supplementary Figure S7 .... Page 8

a) b)

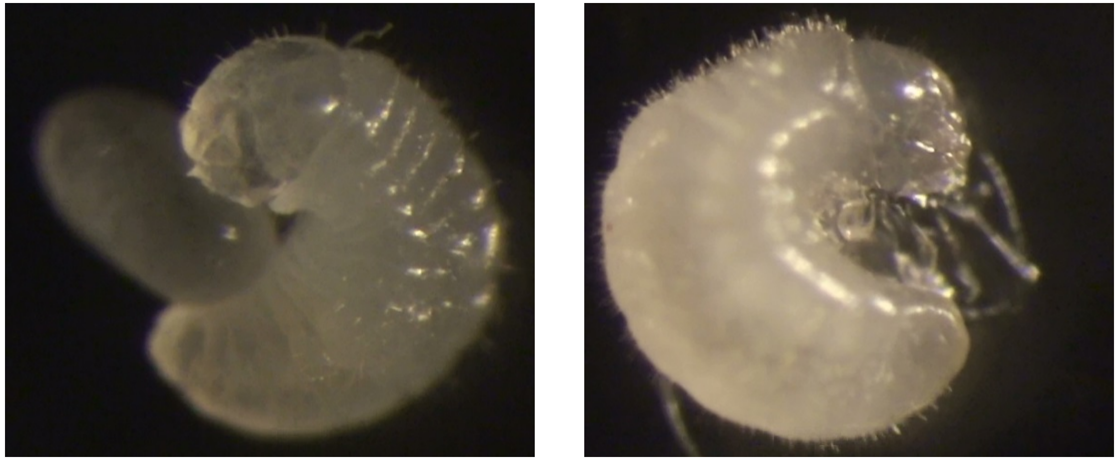

Supplementary Figure S1. *Formica fusca* larva eating an egg. a) before eating the egg b) after eating the egg.

a) b)

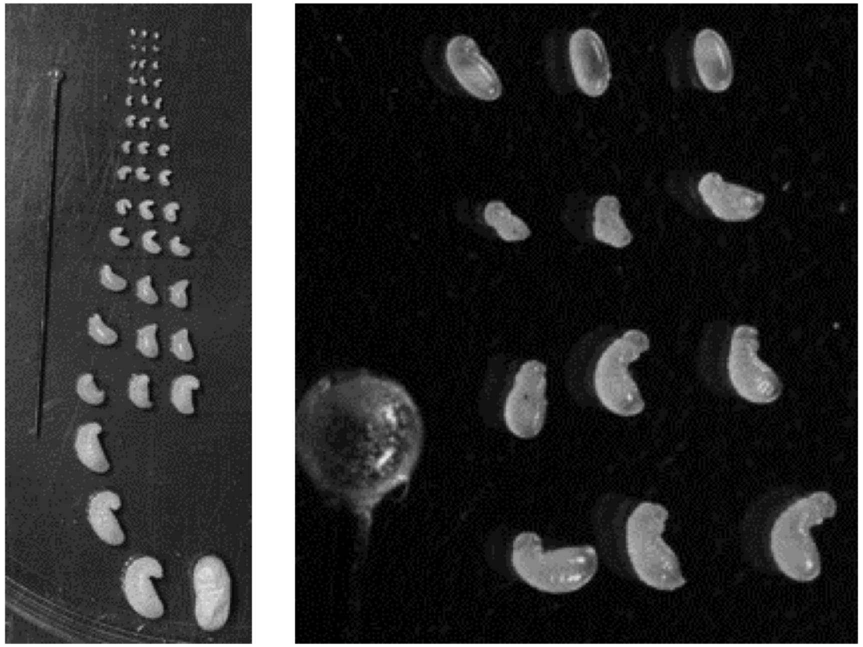

Supplementary Figure S 2. Size of *Formica fusca* larvae used in this study. a) eggs in the top row, pupae in the bottom row, and larvae of increasing size in between b) magnified view of the upper four rows, the bottom two rows representing the larvae used in this study.

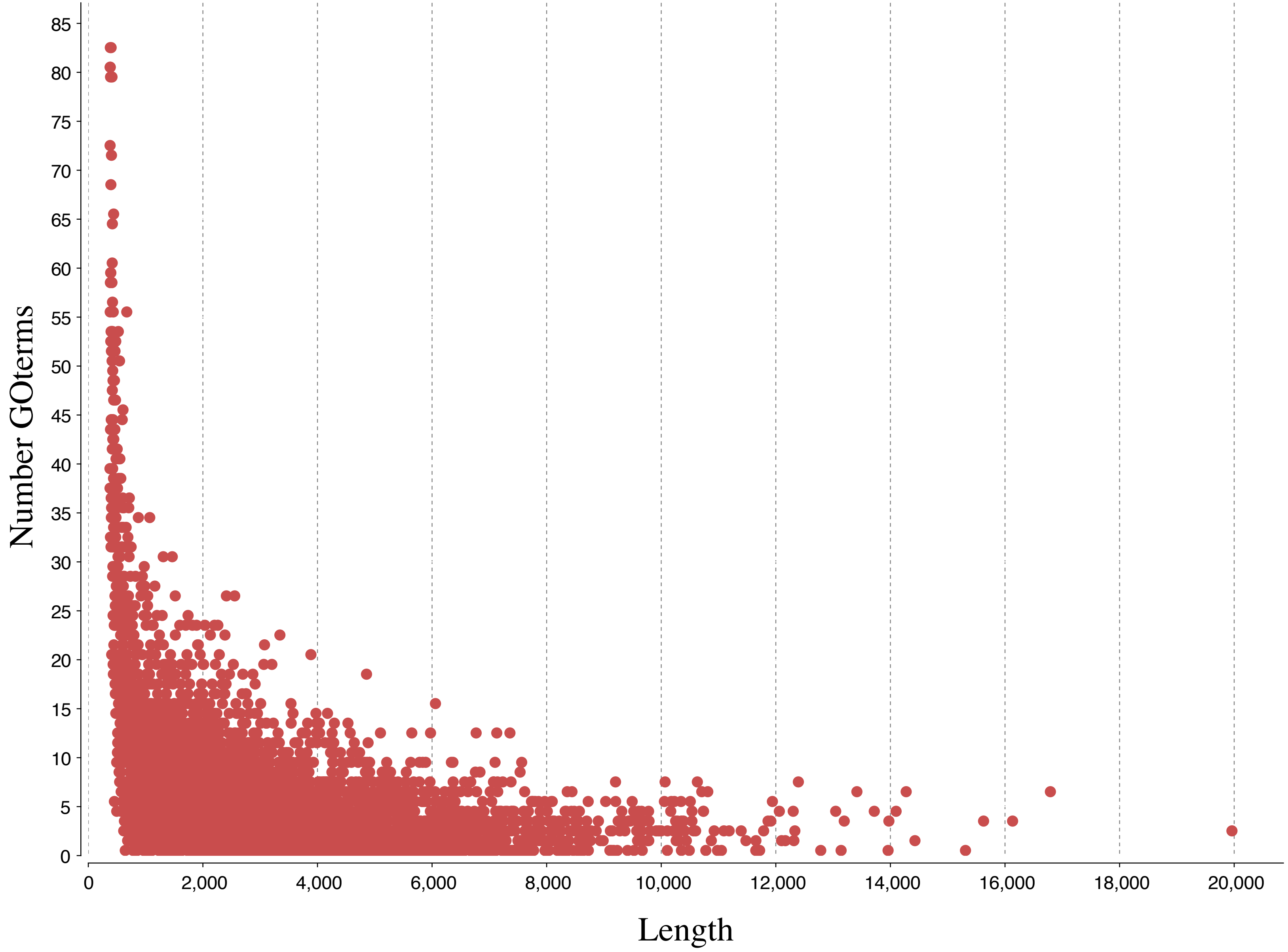

Supplementary Figure S3. Relationship between the length of the unigenes and the number of GOterms associated.

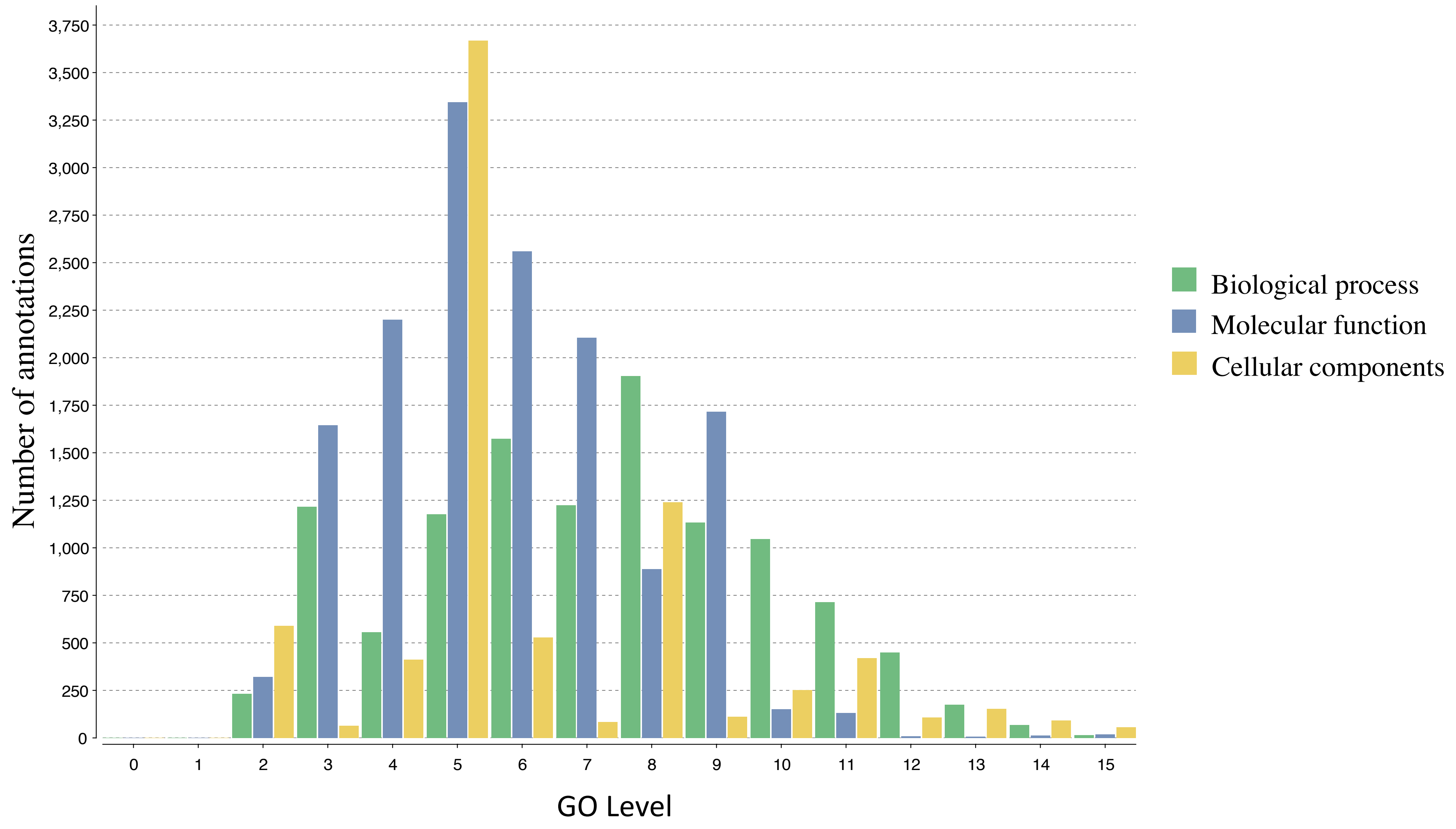

Supplementary Figure S4. Distribution of hierarchical level of GO annotations. For each annotation level, the number of sequences mapping to a term is shown for biological process, molecular function and cellular components. Total annotation = 34402, Mean level of GOterm = 6.36, Deviation = 2.53.

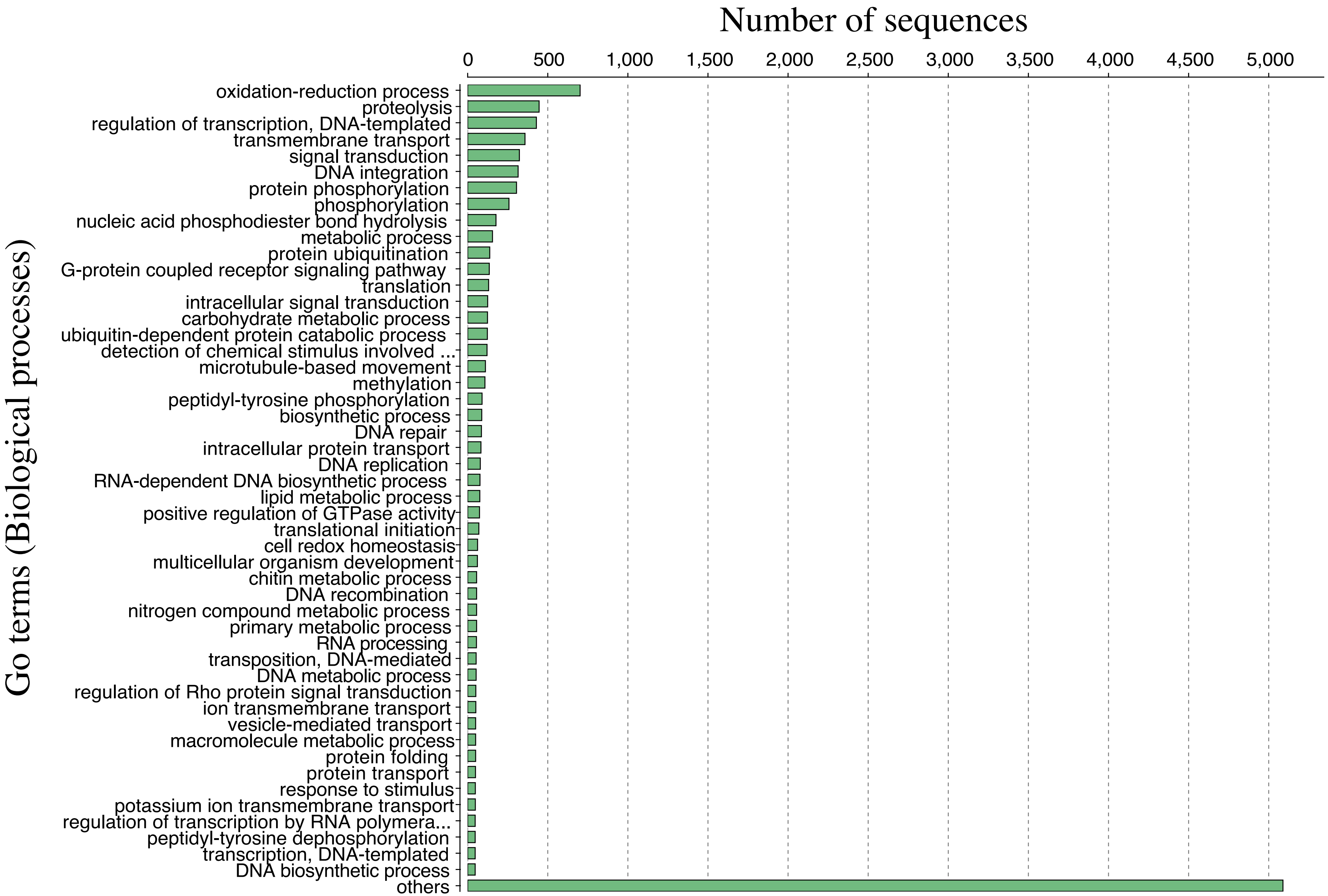

Supplementary Figure S5. GO annotation classification of the top 50 biological processes.

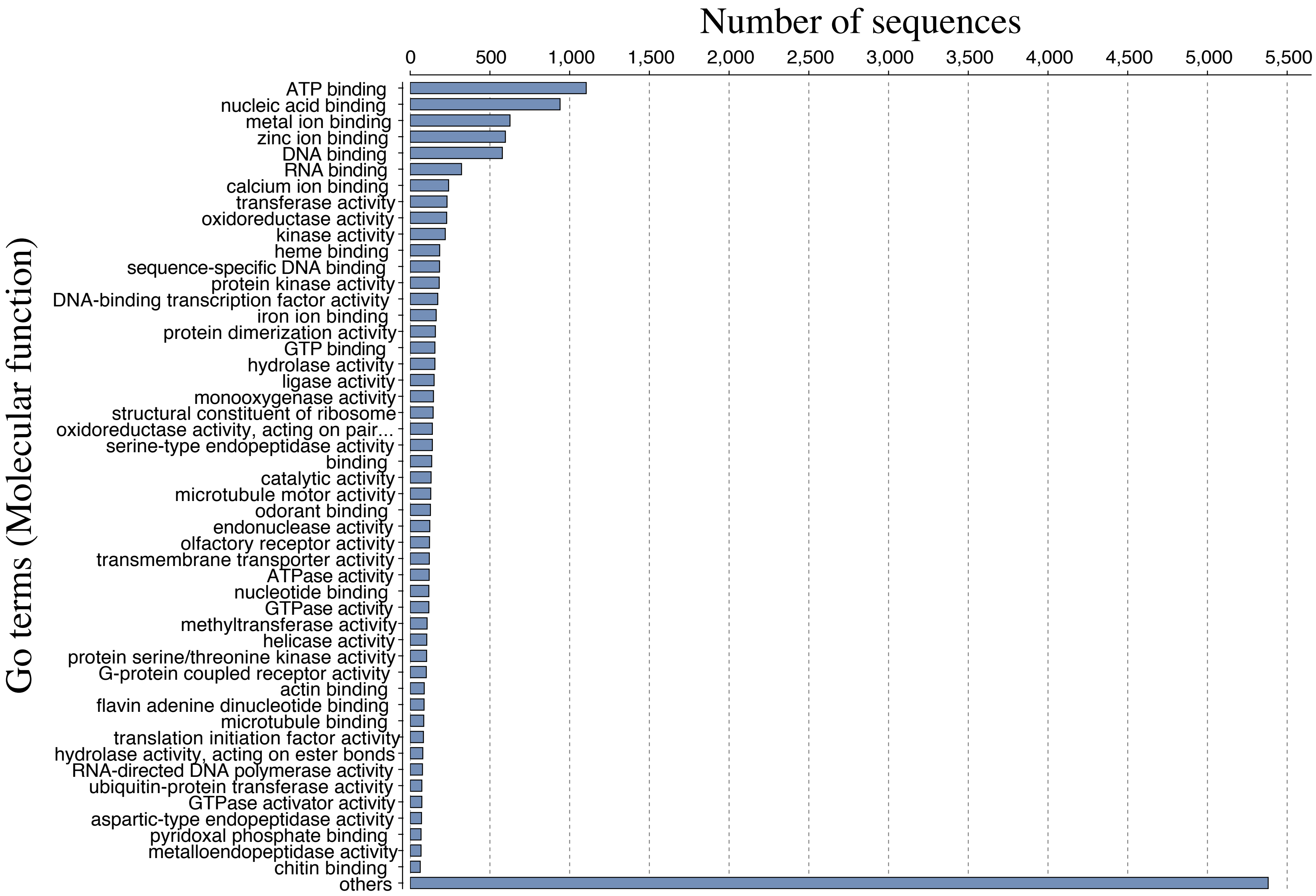

Supplementary Figure S6. GO annotation classification of the top 50 molecular functions.

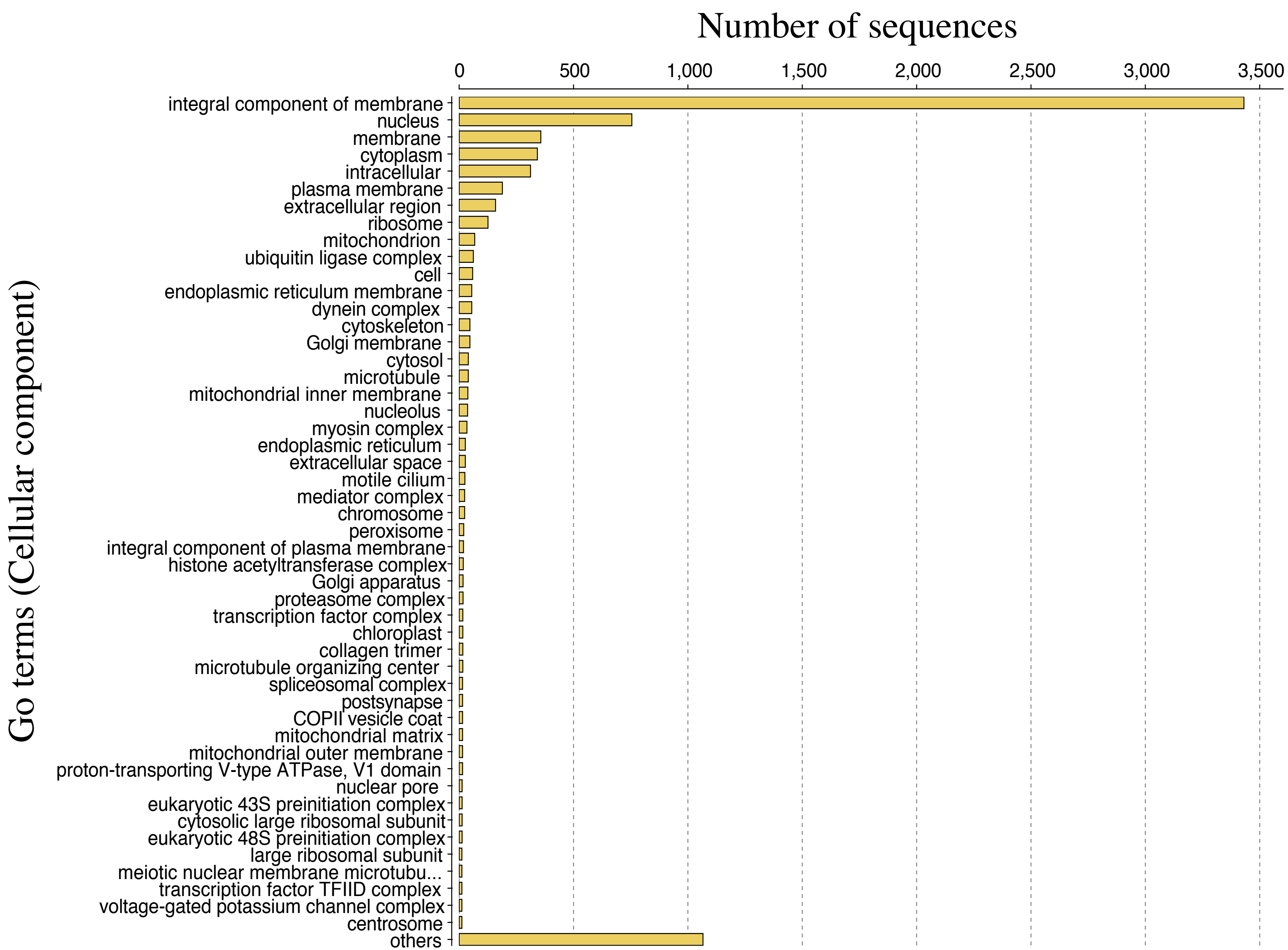

Supplementary Figure S7. GO annotation classification of the top 50 cellular components.
